# The Microbiological Characteristics of Carbapenem-Resistant *Enterobacteriaceae* Carrying the *mcr-1* Gene

**DOI:** 10.3390/jcm8020261

**Published:** 2019-02-19

**Authors:** Chih-Wei Chen, Hung-Jen Tang, Chi-Chung Chen, Ying-Chen Lu, Hung-Jui Chen, Bo-An Su, Tzu-Chieh Weng, Yin-Ching Chuang, Chih-Cheng Lai

**Affiliations:** 1 Division of Neurosurgery, Department of Surgery, Chi Mei Medical Center, Tainan 710, Taiwan; awei921@gmail.com; 2 Department of Occupational Safety and Health/Institute of Industrial Safety and Disaster Prevention, College of Sustainable Environment, Chia Nan University of Pharmacy and Science, Tainan 717, Taiwan; 3 Department of Medicine, Chi Mei Medical Center, Tainan 710, Taiwan; 8409d1@gmail.com (H.-J.T.); uolddy@gmail.com (H.-J.C.); suboan0421@gmail.com (B.-A.S.); wengtzuchieh@gmail.com (T.-C.W.); 4 Department of Medical Research, Chi Mei Medical Center, Tainan 710, Taiwan; ccomm2@yahoo.com.tw (C.-C.C.); chuangkenneth@hotmail.com (Y.-C.C.); 5 Department of Food Science, National Chiayi University, Chiayi 600, Taiwan; biolyc2016@gmail.com; 6 Departments of Medicine, Chi Mei Medical Center, Liouying, Tainan 736, Taiwan; 7 Department of Intensive Care Medicine, Chi Mei Medical Center, Liouying, Tainan 736, Taiwan

**Keywords:** antibiotic resistance, carbapenem-resistant *Enterobacteriaceae*, colistin, *Escherichia coli*, *mcr-1*

## Abstract

Objectives: This study aims to assess the prevalence of the *mcr-1* gene among carbapenem-resistant *Enterobacteriaceae* (CRE) isolated from clinical specimens and to further investigate the clinical significance and microbiological characteristics of CRE carrying the *mcr-1* gene. Methods: Four hundred and twenty-three CRE isolates were screened for the presence of the *mcr-1* gene. After identification, their clinical significance, antibiotic susceptibility, and antibiotic resistance mechanisms including the ESBL gene, carbapenemase gene, outer membrane protein (OMP), and plasmid sequencing were assessed. Results: Only four (0.9%) isolates of carbapenem-resistant *Escherichia coli (E. coli)* were found to carry the *mcr-1* gene and demonstrated different pulsed-field gel electrophoresis (PFGE) patterns and sequence types (ST). While one patient was considered as having *mcr-1*-positive carbapenem-resistant *E. coli* (CREC) colonization, the other three *mcr-1*-positive CREC-related infections were classified as nosocomial infections. Only amikacin and tigecycline showed good in vitro activity against these four isolates, and three of them had a minimum inhibitory concentration with colistin of ≥4 mg/L. In the colistin-susceptible isolate, *mcr-1* was nonfunctional due to the insertion of another gene. In addition, all of the mcr-1-positive CREC contained various resistant genes, such as *AmpC*_CMY_, *bla*_NDM_, *bla*_TEM_, *bla*_SHV_, and *bla*_CTX_. In addition, one strain (EC1037) had loss of the OMP. **Conclusions:** The emergence of the *mcr-1* gene among CRE, especially *E. coli*, remains worth our attention due to its resistance to most antibiotics, and a further national survey is warranted.

## 1. Introduction

In the era of increasing carbapenem resistance among *Enterobacteriaceae*, the treatment options against these drug-resistant pathogens has become limited. Currently, the mainstays of pharmacotherapy against carbapenem-resistant *Enterobacteriaceae* (CRE)-associated infections only include tigecycline, aminoglycosides, and several old antibiotics such as fosfomycin and colistin. *mcr-1* is the first plasmid-mediated colistin resistance gene that can be transferred horizontally via plasmids [1]. Since the first identified *Enterobacteriaceae* carrying a plasmid-encoded colistin-resistance gene *mcr-1* in China [2], more and more sites, including Europe [3,4], Canada [5], Vietnam [6], Hong Kong [7], and Taiwan [8], have reported the presence of *mcr-1*-positive isolates from both animals and humans. The *mcr-1*-positive *Escherichia coli* (*E. coli*) infections are found to be associated with male sex, immunosuppression, and the use of antibiotics, especially for fluoroquinolone and carbapenems. However, most *mcr-1* associated studies are based on the surveillance of colistin-resistant bacteria, and the epidemiology of the *mcr-1* gene among CRE isolates remains unknown [9]. As colistin is one of the limited therapeutic options against CRE, clinicians should be seriously concerned about the colistin resistance mediated by the *mcr-1* gene. Therefore, we conducted this investigation to assess the prevalence of the *mcr-1* gene among CRE isolated from clinical specimens and further investigate the microbiological characteristics of CRE carrying the *mcr-1* gene.

## 2. Methods

### 2.1. Bacterial Isolates

This study was conducted at two medical centers, the Chi Mei Medical Center and the National Cheng Kung University Hospital in southern Taiwan. The bacterial species were confirmed using a VITEK 2 automated system (bioMérieux, Marcy l’Etoile, France) with a VITEK^®^2 GN ID card. These isolates were stored at −80 °C in Protect Bacterial Preservers (Technical Service Consultants Limited, Heywood, UK) before investigation. CRE was defined as resistance to any of four carbapenems (ertapenem, imipenem, doripenem, meropenem). All of the clinical specimens positive for CRE between April 2014 and June 2017 were screened for the presence of the *mcr-1* gene as previously described [10].

### 2.2. Antimicrobial Susceptibility Testing

Standard amikacin, ciprofloxacin, doxycycline, ertapenem, gentamicin, imipenem (U.S. Pharmacopeia, Rockville, MD, USA), ampicillin, cephalothin, cefuroxime, ceftriaxone, ceftazidime, colistin sulfate, doripenem, meropenem, trimethoprim/sulfamethoxazole (Sigma-Aldrich, St. Louis, MO, USA), fosfomycin (Ercros, Barcelona, Spain), and tigecycline (Pfizer, New York, NY, USA) were used for antimicrobial susceptibility testing. The minimum inhibitory concentration (MIC) determinations and susceptibility interpretation criteria followed the Clinical Laboratory and Standard Institute (CLSI) and Federal Drug Administration (FDA) standards [11,12]. The MICs of the drugs, except tigecycline and colistin, were measured by agar dilution in Mueller–Hinton agar (Oxoid, Basingstoke, UK) according to CLSI recommendations.^11^ For fosfomycin susceptibility, glucose-6-phosphate (25 mg/mL) was added to the agar plate. Tigecycline and colistin MICs were determined by microdilutions in freshly prepared cation-adjusted Mueller–Hinton broth (CAMHB). *E. coli* ATCC 25922 was used as the control strain [13]. The MICs of other agents were determined using the custom-designed panels for Gram-negative bacilli (Sensititre, Thermo Fisher Scientific, Oakwood Village, OH, USA).

### 2.3. PCR Detection and Sequencing of Antibiotic Resistance Genes

PCR was used to amplify the ESBL genes (*bla*_TEM_, *bla*_SHV_, *bla*_CTX-M_) and ampC genes (*bla*_DHA-1_ and *bla*_CMY-2_), screening the representative carbapenemase gene (*bla*_KPC-2_, *bla*_NDM_) and *mcr-1* gene using specific primers as previously described [10,14,15,16]. Amplicons of β-lactamase genes were purified with PCR clean-up kits (Roche Diagnostics, GmbH, Penzberg, Germany) and were sequenced on an ABI PRISM 3730 sequencer analyzer (Applied Biosystems, Foster City, CA, USA) [17]. The outer membrane protein (OMP) genes were detected as previously reported [18]. The full-length sequences of ompC and ompF, including their promoter regions, were amplified and sequenced.

### 2.4. Pulsed-Field Gel Electrophoresis (PFGE)

PFGE was performed with a CHEF DR II apparatus (Bio-Rad Laboratories, Hercules, CA, USA) as previously reported [19]. Briefly, DNA was digested by XbaI, and electrophoresis was performed in a 1% agarose gel. The bacteriophage lambda ladder pulsed-field grade (PFG) and low-range PFG molecular weight markers were loaded onto all gels. The similarities of the PFGE profiles of each strain were compared using a Dice coefficient at 1.0% of tolerance and 1.0% of optimization.

### 2.5. Multilocus Sequence Typing (MLST)

MLST was performed with seven housekeeping genes, including *adk*, *fumC*, *gyrB*, *icd*, *mdh*, *purA*, and *recA*. The analysis was conducted as previously described [13,20]. The types of sequences were determined using the MLST database (http://enterobase.warwick.ac.uk/species/index/ecoli).

### 2.6. S1-Nuclease Pulsed Field Gel Electrophoresis (S1-Nuclease PFGE)

Plasmid DNA was extracted from bacteria with the Qiagen Midi Kit (Qiagen). Plasmid sizing was performed using S1-nuclease (Promega) digested plasmid DNA, followed by separation by pulsed field gel electrophoresis (PFGE) using a CHEF mapper system (Bio-Rad, USA) as previously described [21].

### 2.7. Southern Blotting of *mcr-1*

Southern blotting was performed using a semidry transfer system (Bio-Rad) and the *mcr-1*-containing plasmids were identified by hybridization with Dig-labeled *mcr-1*-specific probe generated by the PCR DIG Probe Synthesis Kit, and Detection Starter Kit II (Roche Applied Sciences, Mannheim, Germany) [21].

### 2.8. Plasmid Sequencing

Bacterial pellets in centrifugation tubes were resuspended in buffer. The cell wall was removed by enzymatic digestion in the presence of RNase. Cell lysis and chromosome removal were achieved by alkaline lysis, followed by acid aggregation and centrifugation. DNA in the supernatant was extracted using organic solvent and recovered by ethanol precipitation. Concentration of samples was determined by fluorescence quantification. Purified DNA was analyzed by 0.4% agarose gel electrophoresis. The Illumina MiSeq System (Illumina, San Diego, CA, USA) was used for plasmid sequencing. The derived reads were assembled using the CLC Genomics Workbench 5.51 (CLC bio, Aarhus, Denmark) [22].

## 3. Results

### 3.1. Clinical Significance

**Figure 1:**
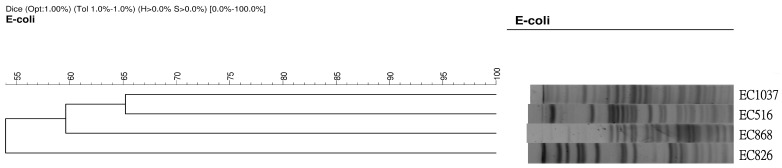
Pulsed-field gel electrophoresis (PFGE) patterns of four *mcr-1*-positive carbapenem-resistant Escherichia coli (*E. coli*).

Among 423 CRE isolates, including *Klebsiella pneumoniae* (*K. pneumoniae)* (*n* = 323), *E. coli* (*n* = 35), *Enterobacter* spp. (*n* = 35), *Citrobacter* spp. (*n* = 17), *Serratia* spp. (*n* = 5), and other bacterial species (*n* = 8), four *E. coli* were found to carry the *mcr-1* gene. All carbapenem-resistant *E. coli* (CREC) exhibited different PFGE patterns (Figure 1). The clinical significance of these four isolates is summarized in Table 1. The age range of the patients was between 54 years and 90 years. Two of the *mcr-1*-positive CREC were isolated from urine specimens, one from ascites, and one from a rectal swab. All of four patients had variable underlying immunocompromised conditions, such as malignancy and chronic kidney disease. In addition, two patients had undergone surgery within one month before the diagnosis of *mcr-1*-positive CREC. All of the patients had received prior broad-spectrum antibiotics including carbapenem, piperacillin/tazobactam, and third-generation cephalosporins. However, none of the patients had previously received colistin. While one patient was considered as having *mcr-1*-positive CREC colonization, the other three *mcr-1*-positive CREC-related infections were classified as nosocomial infections, including two episodes of catheter-associated urinary tract infection and one of peritonitis. Various antibiotic regimens were used for the three cases with infections caused by *mcr-1*-positive CREC. Two of three patients with *mcr-1*-positive CREC infection died in hospital. Only the one patient who had *mcr-1*-positive CREC peritonitis and the patient with *mcr-1*-positive CREC colonization survived. For cases 2 and 4 with survival, *mcr-1*-positive CREC were detected at two and three weeks, respectively. For cases 1 and 3 with mortality, *mcr-1*-positive CREC remained persistent for about one week till the deaths of the patients.

### 3.2. MICs

The MICs of these four isolates are shown in Table 2. For the four *E. coli* isolates, amikacin and tigecycline showed good in vitro activities. Each of the three *E. coli* isolates remained susceptible to fosfomycin and minocycline. In contrast, these four strains showed resistance against the other broad-spectrum antibiotics including cephalosporin, ciprofloxacin, and most carbapenems. Regarding colistin, one isolate (EC826) had an MIC level of only 0.25 mg/L and was classified as colistin wild-type (WT). The other three *E. coli* (EC516, EC868, and EC1037) were identified as colistin nonwild-type (NWT) (MICs ≥4 mg/L).

### 3.3. Molecular Characteristics

**Figure 2:**
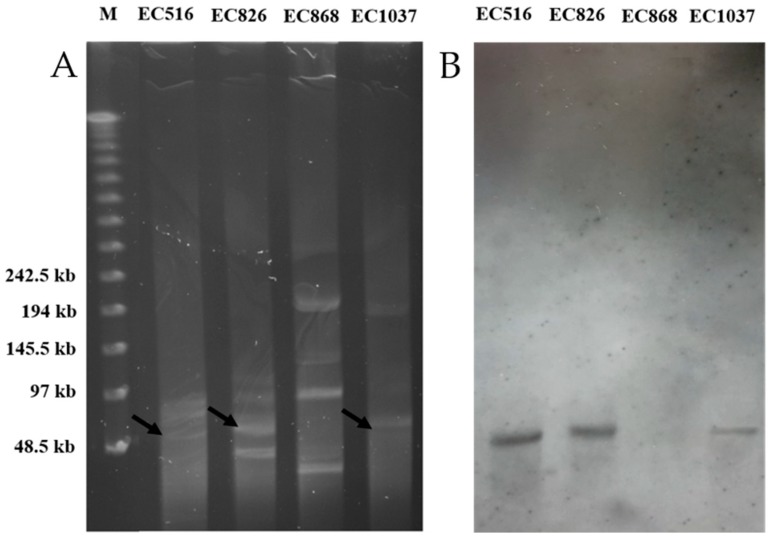
(**A**) S1 nuclease PFGE patterns of plasmids from four clinical isolates. (**B**) the Southern blotting pattern (“M” indicates the lambda molecular weight marker, arrows indicate hybridized plasmids which were shown with positive signals by Southern blot hybridization with the probe).

Based on the MLST, these four strains belonged to different sequence types (ST), EC516 (ST617), EC826 (ST457), EC868 (ST10), and EC103 (ST1196). In this study, we used PCR first to screen and identify *mcr-1* gene, and then we used S1-neculease PFGE and Southern blot of *mcr-1* to help confirm the location of plasmid-mediated *mcr-1* gene and the size of plasmid. Plasmid DNA was linearized by S1 nuclease on PFGE. An agarose gel of the S1 nuclease PFGE-based sizing of plasmids for the four isolates is shown (Figure 2A). Figure 2B shows the corresponding gel after Southern blotting, and the plasmids expressing *mcr-1* were detected by hybridization of the Southern blot with a specific probe. The findings of the Southern blot indicated that the *mcr-1* gene was found in the plasmids of EC516, EC826, and EC1037. However, it was not found in the plasmids of EC868 (Figure 2B)

### 3.4. Antibiotic Resistance Mechanism

Table 3 lists the antibiotic resistance genes for the four *mcr-1*-positive CRE isolates. One strain (EC1037) had *bla*_SHV_, which is closely matched with SHV-5 with only one mutation at L31Q. Three of them carried *bla*_CMY-2_ genes and one had the *bla*_TEM-1_ gene. In addition, one *E coli* isolate (EC516) carried *bla*_CTX-M-65_ and *bla*_NDM-9_. None of them had IMP, VIM, KCP, and OXA48 genes. Regarding the outer membrane proteins, OmpC and OmpF were detected in three of the isolates. Some of them were wild type (EC516 and 868) and one (EC826) was an insertion type. Only one mutation G181D of OmpC was found in EC868 (Table 3). OmpC and OmpF were nondetectable in EC1037.

### 3.5. Sequencing Analyses of the Carbapenem-Resistance Plasmids

**Figure 3:**
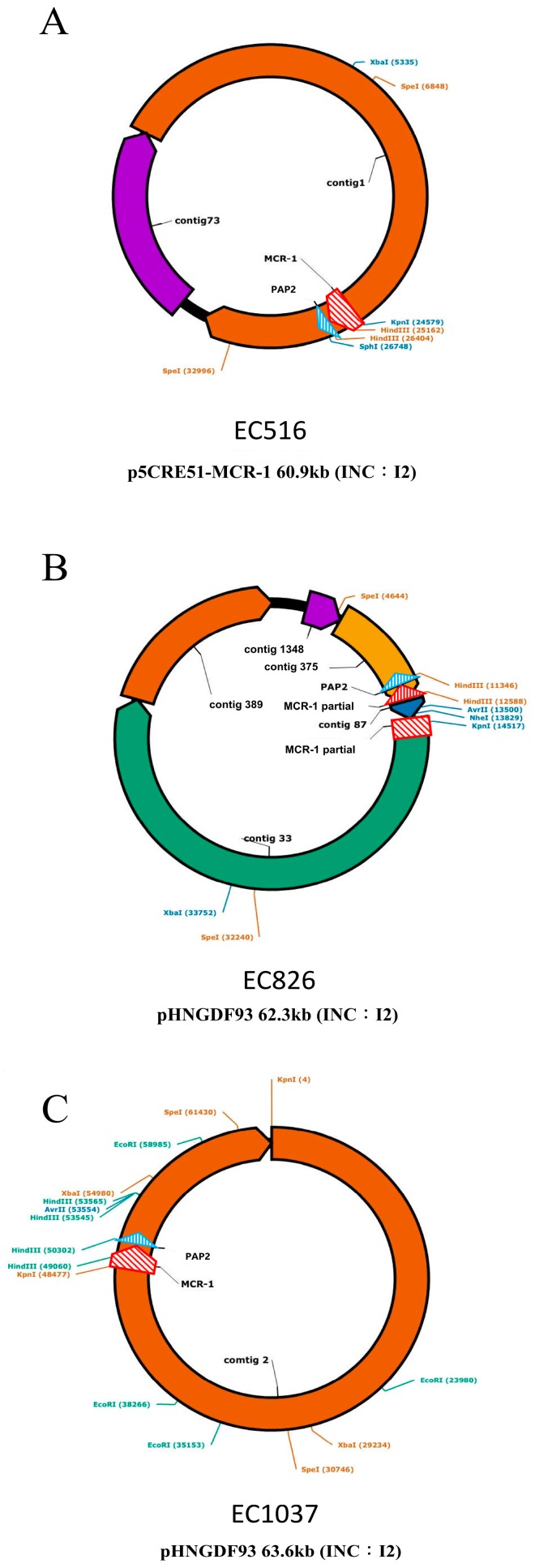
Schematic diagrams of plasmids (**A**) EC516 (p5CRE51-MCR-1), (**B**) EC826 (pHNGDF93), and (**C**) EC1037 (pHNGDF93).

All of the sequencing analyses of carbapenem-resistance plasmids are shown in Figure 3. For *E. coli* EC516, its *bla*_mcr-1_-encoding plasmid was p5CRE51-MCR-1, with a size of approximately 60.9 kb. For *E. coli* EC826, its *bla*_mcr-1_-encoding plasmid was pHNGDF93, with a size of approximately 62.3 kb containing one insertion within *mcr-1*. For *E. coli* EC1037, its main *bla*_mcr-1_-encoding plasmid was pHNGDF93 with a size of approximately 63.6. Only one CRCE 868 isolate with *mcr-1* inserted in the chromosome, not within the plasmid, was found.

## 4. Discussion

In this multicenter study, the overall prevalence of *mcr-1* among CRE was 0.9% (4/423), and all of the four CRE harboring *mcr-1* were *E. coli*. Therefore, the prevalence of *mcr-1* among 35 CREC in this surveillance study was 11.4%. In addition, all of them exhibited different PFGE patterns and ST types, which indicated that they did not result from the spread of a single clone. In China, a national surveillance study [23] of 1105 CRE isolates showed that two CREC carried the *mcr-1* gene and that the prevalence was less than 0.2%. Another surveillance study [24] at a single center in China revealed a similar finding in that only one *E. coli* strain among 1311 CRE isolates harbored *mcr-1*. Although all of these findings indicate that the prevalence of the *mcr-1* gene among CRE isolates remains low, further regular surveillance investigation is still needed to assess the epidemiology of *mcr-1* among CRE isolates. Moreover, the emergence of mcr-1 among CREC should be closely monitored.

In this study, all of the patients with *mcr-1* CRE had various underlying diseases, and two of them had undergone abdominal surgery recently. Although all of them had received broad-spectrum antimicrobial agents, especially piperacillin/tazobactam before acquiring *mcr-1* CRE, none of them had received colistin recently. The clinical manifestations of patients infected or colonized with *mcr-1* CRE in this study were consistent with those of a previous report [8], and patients with multiple comorbidity and history of broad-spectrum antibiotic might be at risk of acquiring *mcr-1*-positive *Enterobacteriaceae*. In line with previous reports [9,25], our study shows that prior use of colistin may not be necessary

In agreement with a previous study [24], all of *mcr-1*-positive CREC in this study displayed resistance against most of the antibiotics. Only minocycline, tigecycline, amikacin, and fosfomycin showed good in vitro activities against more than three CREC strains. This finding and previous reports [26,27] about CRE isolates suggested that combination therapy or some active agents, such as tigecycline, fosfomycin, and amikacin, may be appropriate options for antibiotic treatment. However, a further large-scale study is needed to assess the activity of antibiotics against *mcr-1* gene harboring CRE.

In this study, all of *mcr-1*-positive CREC had resistance genes *AmpC*_CMY_, *bla*_NDM_, *bla*_TEM_, *bla*_SHV_, and *bla*_CTX_. In addition, some of them (EC516, EC826 and EC868) had the detectable outer membrane proteins OmpF and OmpC. However, one strain (EC1037) had a loss of the outer membrane protein. This phenomenon has been observed in previous studies in Taiwan [13,28,29]. The coexistence of plasmidic *AmpC* β-lactamase and outer membrane protein loss could be the main underlying mechanism for non-carbapenem-susceptible *Enterobacteriaceae* [12,24].

Generally, bacteria with the gene encoding plasmid-mediated colistin resistance (*mcr-1*) would be considered as colistin NWT. However, one recent study [9] showed that two (3%) of 76 *mcr-1*-positive *E. coli* remained susceptible to colistin. In this study, one isolate (EC826) was found to have *mcr-1* during the screening, but its colistin MIC was only 0.25 mg/L. We found that another gene was inserted in *mcr-1* in EC826. This finding may explain why the CRE with *mcr-1* demonstrated a susceptible colistin MIC due to the malfunction of *mcr-1*. A similar finding was reported by Terveer et al., where *mcr-1*-positive *E. coli* was phenotypically susceptible to colistin with a MIC of <0.25mg/L, due to a 1329-bp transposon IS10R inserted into the *mcr-1* gene [30]. However, a further large-scale study is warranted to clarify this issue.

Although only 35 (8.2%) clinical isolates in this surveillance of 423 CRE belonged to *E. coli*, all of *mcr-1*-positive CRE were exclusively *E. coli*. In contrast, *K. pneumoniae* (*n* = 323, 76.4%) was the most common CRE in this study, but none of them were found to carry the *mcr-1* gene. Several large surveillance studies demonstrated that the prevalence of *mcr-1* gene among *E. coli* isolates varied, at 0.25% (10/3902) in Italy [31], and 1% (76/5332) in China [9]. For ESBL *E. coli*, the prevalence of the *mcr-1* gene was 3.5% (25/706) in China [25], but for CREC, only limited reports [23,24,32,33] showed the co-presence of the *mcr-1* gene in human and animal isolates. For this study, the sample size of CREC is small, and thus may result in the high prevalence of *mcr-1* gene among CREC isolates (11.4%, 4/35). A further large-scale study is needed to investigate the prevalence of *mcr-1* gene among CREC isolates.

In conclusion, the emergence of the *mcr-1* gene among CRE, especially *E. coli* in southern Taiwan, remains worth our attention due to its resistance to most antibiotics. Although the *E. coli* harboring *mcr-1* gene is rarely susceptible to colistin, a further national survey is warranted.

## Figures and Tables

**Table 1:** Clinical significance of *mcr-1*-positive carbapenem-resistant *E. coli* isolates.

Case Number (year)	Specimen	Species	Age/Sex	Underlying Diseases	Recent Surgery within One Month	Type of Infection	Previous Antibiotic Use in the Past Month	Treatment	Outcome
1 (2016)	Urine	*E. coli* (EC516)	79/F	Colon cancer with multiple metastasis, chronic kidney diseases	Colon cancer post operation	CAUTI	Piperacillin/tazobactam, cefazolin, gentamicin, metronidazole	Piperacillin/tazobactam	Death
2 (2016)	Urine	*E. coli* (EC826)	54/M	End-stage renal disease, enterovesical fistula	Nil	CAUTI	Ertapenem, doripenem, piperacillin/tazobactam, flomoxef	Colistin + fosfomycin	Survival
3 (2016)	Ascites	*E. coli* (EC868)	68/M	Cholangiocarcinoma	Subtotal gastrectomy	Peritonitis	Piperacillin/tazobactam, meropenem	Ceftazidime	Death
4 (2017)	Rectal swab	*E. coli* (EC1037)	90/F	Bronchiectasis, chronic kidney disease, chronic respiratory failure	Nil	Colonization	Piperacillin/tazobactam, ceftazidime, flomoxef	Nil	Survival

CAUTI: catheter-associated urinary tract infection; *E: coli: Escherichia coli*.

**Table 2:** MIC results of *mcr-1*-positive carbapenem-resistant *E. coli* isolates.

	EC516	EC826	EC868	EC1037
Amikacin	2	2	4	4
Cefazolin	**>128**	**>128**	**>128**	**>128**
Cefmetazole	**>128**	**>128**	**>128**	**>128**
Cefotaxime	**>128**	**>128**	**>128**	**>128**
Cefepime	**>128**	**16**	**32**	**128**
Ciprofloxacin	**64**	**64**	**8**	**>128**
Doripenem	**8**	**4**	**8**	2
Ertapenem	**16**	**128**	**32**	**>32**
Imipenem	**8**	**32**	**8**	**4**
Meropenem	**4**	**8**	**8**	**2**
Fosfomycin	**1024**	4	1	4
Gentamicin	**32**	**2**	**128**	**>128**
Minocycline	8	1	2	**>128**
Tigecycline	0.25	0.5	1	1
Colistin	**16**	0.25	**16**	**4**

Bold type indicates resistance.

**Table 3:** Antibiotic resistance mechanisms of *mcr-1*-positive *Enterobacteriaceae* isolates.

Isolates	EC516	EC826	EC868	EC1037
Resistance gene				
mcr	1	1 (insertion)	1	1
SHV	-	-	-	Close match SHV-5 (L31Q)
DHA	-	-		
CMY	-	2	2	2
TEM	-	-	-	1
CTX-M	65	-	-	
NDM	9	-	-	
IMP	-	-	-	
VIM	-	-	-	
KPC	-	-	-	
OXA48	-	-	-	-
Outer membrane protein profiles				
OmpC	Wild type	Insertion	Mutation (G181D)	Non detected
OmpF	Wild type	Insertion	Wild type	Non detected

## References

[1] Perez F., El Chakhtoura N.G., Papp-Wallace K., Wilson B.M., Bonomo R.A. (2016). Treatment options for infections caused by carbapenem-resistant Enterobacteriaceae: Can we apply “precision medicine” to antimicrobial chemotherapy?. Expert. Opin. Pharmacother..

[2] Liu Y.Y., Wang Y., Walsh T.R., Yi L.X., Zhang R., Spencer J., Doi Y., Tian G., Dong B., Huang X. (2016). Emergence of plasmid-mediated colistin resistance mechanism MCR-1 in animals and human beings in China: A microbiological and molecular biological study. Lancet Infect. Dis..

[3] Brennan E., Martins M., McCusker M.P., Wang J., Alves B.M., Hurley D., El Garch F., Woehrlé F., Miossec C., McGrath L. (2016). Multidrug-resistant Escherichia coli in Bovine animals, Europe. Emerg. Infect. Dis..

[4] Falgenhauer L., Waezsada S.E., Gwozdzinski K., Ghosh H., Doijad S., Bunk B., Spröer C., Imirzalioglu C., Seifert H., Irrgang A. (2016). Chromosomal locations of mcr-1 and BLA CTX-M-15 in fluoroquinolone-qesistant Escherichia coli ST410. Emerg. Infect. Dis..

[5] Payne M., Croxen M.A., Lee T.D., Mayson B., Champagne S., Leung V., Bariso S., Hoang L., Lowe C. (2016). Mcr-1-positive colistin-resistant Escherichia coli in traveler returning to Canada from China. Emerg. Infect. Dis..

[6] Malhotra-Kumar S., Xavier B.B., Das A.J., Lammens C., Hoang H.T., Pham N.T., Goossens H. (2016). Colistin-resistant Escherichia coli harbouring mcr-1 isolated from food animals in Hanoi, Vietnam. Lancet Infect. Dis..

[7] Wong S.C., Tse H., Chen J.H., Cheng V.C., Ho P.L., Yuen K.Y. (2016). Colistin-resistant Enterobacteriaceae carrying the mcr-1 gene among Patients in Hong Kong. Emerg. Infect. Dis..

[8] Lai C.C., Lin Y.T., Lin Y.T., Lu M.C., Shi Z.Y., Chen Y.S., Wang L.S., Tseng S.H., Lin C.N., Chen Y.H. (2018). Clinical characteristics of patients with bacteraemia due to the emergence of mcr-1-harbouring Enterobacteriaceae in humans and pigs in Taiwan. Int. J. Antimicrob. Agents.

[9] Wang Y., Tian G.B., Zhang R., Shen Y., Tyrrell J.M., Huang X., Zhou H., Lei L., Li H.Y., Doi Y. (2017). Prevalence, risk factors, outcomes, and molecular epidemiology of mcr-1-positive Enterobacteriaceae in patients and healthy adults from China: An epidemiological and clinical study. Lancet Infect. Dis..

[10] Ye H., Li Y., Li Z., Gao R., Zhang H., Wen R., Gao G.F., Hu Q., Feng Y. (2016). Diversified mcr-1-harbouring plasmid reservoirs confer resistance to colistin in human gut microbiota. MBio.

[11] Clinical and Laboratory Standards Institute (2018). Performance Standards for Antimicrobial Susceptibility Testing: 28th Informational Supplement.

[12] Brown S.D., Traczewski M.M. (2007). Comparative in vitro antimicrobial activity of tigecycline, a new glycylcycline compound, in freshly prepared medium and quality control. J. Clin. Microbiol..

[13] Ma L., Siu L.K., Lin J.C., Wu T.L., Fung C.P., Wang J.T., Lu P.L., Chuang Y.C. (2013). Updated molecular epidemiology of carbapenem-non-susceptible Escherichia coli in Taiwan: First identification of KPC-2 or NDM-1-producing *E. coli* in Taiwan. BMC Infect. Dis..

[14] Tang H.J., Ku Y.H., Lee M.F. (2015). In vitro activity of imipenem and colistin against a carbapenem-resistant Klebsiella pneumoniae isolate coproducing SHV-31, CMY-2, and DHA-1. Biomed. Res. Int..

[15] Eckert C., Gautier V., Saladin-Allard M., Hidri N., Verdet C., Ould-Hocine Z., Barnaud G., Delisle F., Rossier A., Lambert T. (2004). Dissemination of CTX-M-type beta-lactamases among clinical isolates of Enterobacteriaceae in Paris, France. Antimicrob. Agents Chemother..

[16] Queenan A.M., Bush K. (2007). Carbapenemases: The versatile beta-lactamases. Clin. Microbiol. Rev..

[17] Diancourt L., Passet V., Verhoef J., Grimont P.A., Brisse S. (2005). Multilocus sequence typing of Klebsiella pneumoniae nosocomial isolates. J. Clin. Microbiol..

[18] Yan J.J., Wu J.J., Lee C.C., Ko W.C., Yang F.C. (2010). Prevalence and characteristics of ertapenem-nonsusceptible Escherichia coli in a Taiwanese university hospital, 1999 to 2007. Eur. J. Clin. Microbiol. Infect. Dis..

[19] Lai C.C., Chen C.C., Hung H.L., Chuang Y.C., Tang H.J. (2016). The role of doxycycline in the therapy of multidrug-resistant *E. coli*—An in vitro study. Sci. Rep..

[20] Lai C.C., Chen C.C., Lu Y.C., Chen H.J., Su B.A., Weng T.C., Chiu Y.H., Toh H.S., Zhang C.C., Chiang S.R. (2018). Simultaneous three Enterobacteriaceae with different bla NDM-1-encoding plasmids in a patient transferred from mainland China to Taiwan. Infect. Drug Resist..

[21] Mshana S.E., Hain T., Domann E., Lyamuya E.F., Chakraborty T., Imirzalioglu C. (2013). Predominance of Klebsiella pneumoniae ST14 carrying CTX-M-15 causing neonatal sepsis in Tanzania. BMC Infect. Dis..

[22] Scott D., Ely B. (2015). Comparison of genome sequencing technology and assembly methods for the analysis of a GC-rich bacterial genome. Curr. Microbiol..

[23] Zhang R., Liu L., Zhou H., Chan E.W., Li J., Fang Y., Li Y., Liao K., Chen S. (2017). Nationwide Surveillance of Clinical Carbapenem-resistant Enterobacteriaceae (CRE) Strains in China. EBioMedicine.

[24] Li Y., Sun Q.L., Shen Y., Zhang Y., Yang J.W., Shu L.B., Zhou H.W., Wang Y., Wang B., Zhang R. (2018). Rapid Increase in Prevalence of Carbapenem-Resistant Enterobacteriaceae (CRE) and Emergence of Colistin Resistance Gene mcr-1 in CRE in a Hospital in Henan China. J. Clin. Microbiol..

[25] Bi Z., Berglund B., Sun Q., Zhang Y., Yang J.W., Shu L.B., Zhou H.W., Wang Y., Wang B., Zhang R. (2017). Prevalence of the mcr-1 colistin resistance gene in extended-spectrum β-lactamase-producing *Escherichia coli* from human faecal samples collected in 2012 in rural villages in Shandong Province, China. Int. J. Antimicrob. Agents..

[26] Trecarichi E.M., Tumbarello M. (2017). Therapeutic options for carbapenem-resistant Enterobacteriaceae infections. Virulence.

[27] Rodriguez-Bano J., Gutierrez-Gutierrez B., Machuca I., Pascual A. (2018). Treatment of infections caused by extended-spectrum-beta-lactamase-, ampC-, and carbapenemase-producing Enterobacteriaceae. Clin. Microbiol. Rev..

[28] Poirel L., Heritier C., Spicq C., Pascual A. (2004). In vivo acquisition of high-level resistance to imipenem in *Escherichia coli*. J. Clin. Microbiol..

[29] Chia J.H., Siu L.K., Su L.H., Lin H.S., Kuo A.J., Lee M.H., Wu T.L. (2009). Emergence of carbapenem-resistant *Escherichia coli* in Taiwan: Resistance due to combined CMY-2 production and porin deficiency. J. Chemother..

[30] Terveer E.M., Nijhuis R.H.T., Crobach M.J.T., Knetsch C.W., Veldkamp K.E., Gooskens J., Kuijper E.J., Claas E.C.J. (2017). Prevalence of colistin resistance gene (mcr-1) containing Enterobacteriaceae in feces of patients attending a tertiary care hospital and detection of a mcr-1 containing, colistin susceptible *E. coli*. PLoS ONE.

[31] Principe L., Piazza A., Mauri C., Anesi A., Bracco S., Brigante G., Casari E., Agrappi C., Caltagirone M., Novazzi F. (2018). Multicenter prospective study on the prevalence of colistin resistance in *Escherichia coli*: Relevance of mcr-1-positive clinical isolates in Lombardy, Northern Italy. Infect. Drug Resist..

[32] Wang R., Liu Y., Zhang Q., Jin L., Wang Q., Zhang Y., Wang X., Hu M., Li L., Qi J. (2018). The prevalence of colistin resistance in *Escherichia coli* and Klebsiella pneumoniae isolated from food animals in China: Coexistence of mcr-1 and blaNDM with low fitness cost. Int. J. Antimicrob. Agents.

[33] Huang T.D., Bogaerts P., Berhin C., Hoebeke M., Bauraing C., Glupczynski Y., A multicentre study group (2017). Increasing proportion of carbapenemase-producing Enterobacteriaceae and emergence of a MCR-1 producer through a multicentric study among hospital-based and private laboratories in Belgium from September to November 2015. Euro. Surveill..

